# Identifying microbial functional guilds performing cryptic organotrophic and lithotrophic redox cycles in anaerobic granular biofilms

**DOI:** 10.1371/journal.pone.0330380

**Published:** 2025-08-18

**Authors:** Zachary Flinkstrom, Samuel J. Bryson, Bojan Pelivano, Pieter Candry, Kristopher A. Hunt, Mari-Karoliina H. Winkler

**Affiliations:** 1 Department of Civil and Environmental Engineering, University of Washington, Seattle, Washington, United States of America; 2 Phase Genomics, Seattle, Washington, United States of America; 3 Laboratory of Systems and Synthetic Biology, Wageningen University & Research WE, Wageningen, The Netherlands; Friedrich Schiller University, GERMANY

## Abstract

Granular biofilms used in anaerobic digester systems contain diverse microbial populations that interact to hydrolyze organic matter and produce methane within controlled environments. Prior research investigated the feasibility of utilizing granular biofilms obtained from an anaerobic digester to remove nitrate without the addition of exogenous electron donors. These granules possessed a unique structure of alternating light and dark iron sulfide and pyrite rich layers that potentially served as both an electron source and sink, linking carbon, nitrogen, sulfur, and iron cycles. To characterize the functional roles of diverse microbial populations enriched within these layered biofilms, we analyzed metagenomes obtained from three different granules. Comparisons between the functional gene content of forty metagenome assembled genomes (MAGs) identified phylogenetically cohesive functional guilds. Each of these functional MAG clusters was assigned to specific steps in anaerobic digestion (hydrolysis, acidogenesis, acetogenesis, and methanogenesis) and anaerobic respiration (denitrification and sulfate reduction). Comparisons with metagenomes derived from a variety of natural and engineered ecosystems confirmed that the enriched denitrifying bacteria were similar to populations typically found in wetlands and biological nitrogen removal systems. Analysis of read alignments to individual genes within the forty MAGs identified conserved genomic features that were representative of the functions that distinguished functional guilds. Overall, this research illustrates the utility of functional based classification of microorganisms for characterizing ecosystem functions and highlights the potential application of engineered ecosystems to serve as experimental models for complex natural ecosystems.

## Introduction

Granular biofilms cultivated within engineered ecosystems have been harnessed for several applications; first in anaerobic digesters that treat organic rich waste streams and produce biogas [1–3], and later for biological nitrogen removal from wastewater [4–6]. Anaerobic digester systems employ microbial populations that carry out sequential and interacting processes that govern the degradation of organic matter: hydrolysis, acidogenesis, acetogenesis, and methanogenesis [1]. Beyond engineering applications, the microbial processes that anaerobically degrade organic matter and produce methane are found in diverse anoxic environments, such as the rumen [7] and wetland soils [8], suggesting that engineered ecosystems may serve as experimentally tractable models to better understand microbial processes occurring in natural ecosystems.

Much of the research into anaerobic digester microbial communities has focused on improved process engineering [9] and the development of predictive mathematical models [10–12]. More recently, the application of molecular methods to study the microbial communities of anaerobic digesters has focused on characterizing the community composition and identifying functional roles of specific taxa [13,14]. Studies have utilized metagenomic sequencing and analysis of recovered genomes to identify core microbial functional guilds, which are sets of organisms with similar metabolic capacities, and understand the contributions of individual taxa to the anaerobic digester metabolic networks [15,16]. Furthermore, molecular approaches have been harnessed to examine impacts of operational controls such as temperature [17], granule size [18], and substrate dynamics [19] – linking performance and microbial community composition.

Different types of waste streams derived from agricultural, municipal, or more specialized waste products impose strong selective pressures [20] that result in reduced taxonomic diversity compared to natural ecosystems [21,22]. Additionally, bioreactors and individual biofilm granules represent defined system boundaries, whereas natural ecosystems can be investigated at different scales ranging from individual soil aggregates [23] to more broadly defined regions such as forests, wetlands, agricultural soils, and sediments [24]. These attributes make engineered ecosystems tractable model systems to investigate more generalized questions in microbial ecology and evolution.

**Fig 1:**
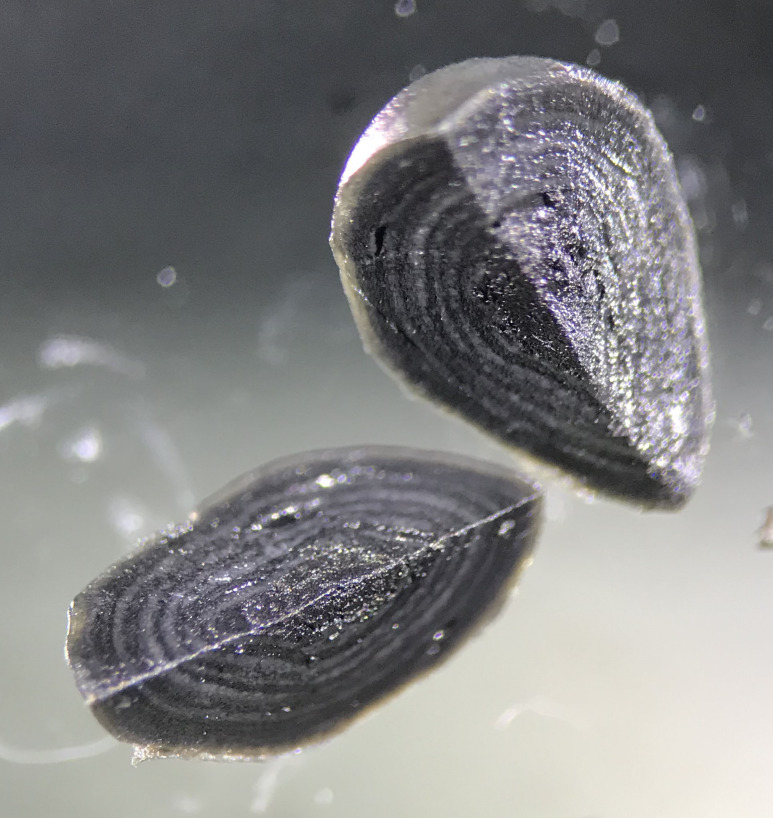
Layered structure of granular biofilms. Anaerobic granule sliced showing alternating light and dark layers. Granule sizes were typically in the 3 to 5 mm diameter range.

Investigations of anaerobic digesters have been useful for understanding the mechanisms and microbial interactions that facilitate organic matter degradation and methanogenesis. Studying how fermentative and methanogenic microorganisms interact with nitrate-, sulfate-, or iron-respiring microbes found in terrestrial and aquatic ecosystems requires the development of engineered systems that support these ecological processes. An example of this can be found in previous research that investigated the feasibility of utilizing granular biofilms obtained from a full-scale upflow anaerobic sludge blanket reactor to treat nitrate polluted water [25]. Dissection of these granules revealed a surprisingly well differentiated layered structure, consisting of alternating light and dark zones (Fig 1). Batch tests performed on these granules demonstrated active methanogens, sulfate reducers, and nitrate reducers that competed for acetate, and revealed a biofilm community that was powered by electron donors generated through hydrolysis and fermentation of endogenous organic matter [25]. Additionally, the observation of iron sulfide (FeS) and pyrite (FeS₂) in the biofilm indicated the potential role of cryptic iron and sulfur redox cycles—closed loops of rapid oxidation and reduction reactions that leave little net change in measurable redox species but can strongly influence microbial metabolism and mineral formation. Such cryptic cycles have been documented in marine sediments, where they drive coupled biotic and abiotic transformations that shape sediment geochemistry [26]. Taken together, these characteristics suggest that anaerobic granular biofilms cultured with the addition of alternative electron acceptors (e.g., nitrate, sulfate, or iron) may serve as a model experimental system for investigating the responses of natural ecosystems to perturbations in redox state.

This study addressed the research gap of whether clustering metagenome assembled genomes (MAGs) by functional gene content could be used to define meaningful functional guilds within complex anaerobic communities, and whether these guilds—and the taxa that comprise them—are relevant beyond a single engineered system to other anaerobic ecosystems. To fill this gap, we performed short (Illumina) and long-read (Oxford Nanopore) sequencing, assembly, genome reconstruction, annotation, and functional analysis. The functional repertoire of MAGs was clustered to identify functional guilds performing specific redox functions. We then compared the metagenomes and recovered MAGs to publicly available metagenomes derived from different environments including anaerobic digesters, activated sludge, bovine rumen, landfill leachate, wetland sediments, and groundwater which revealed the organisms and functions conserved across these disparate ecosystems.

## Results

### Methanogens are the most abundant granule community members

**Fig 2:**
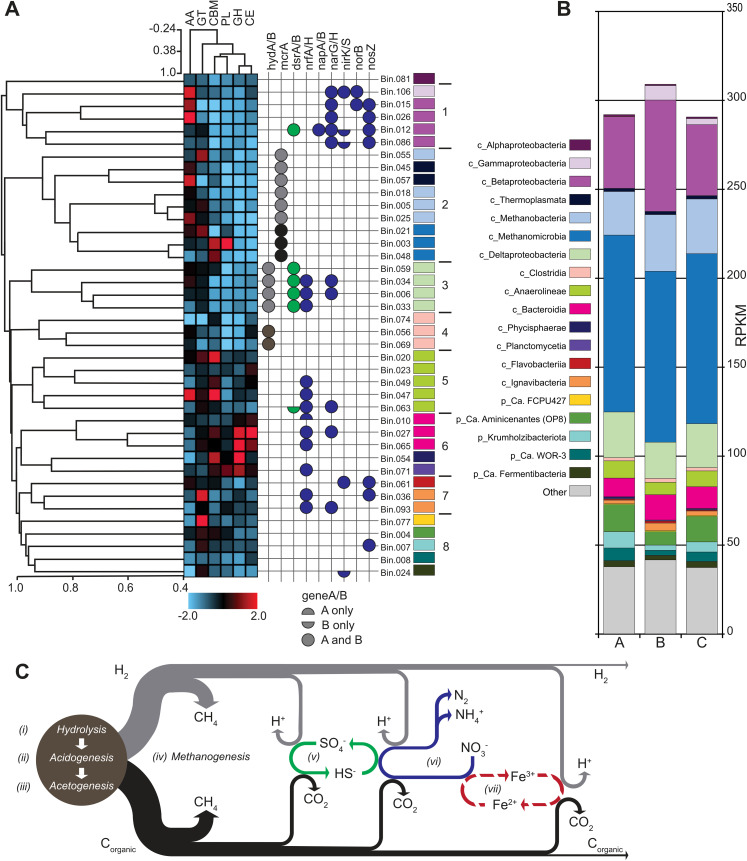
Functional and taxonomic characterization of granular biofilms. (**A** left to right) The dendrogram presents clustering based on Pearson correlation of normalized KEGG ortholog (KO) annotation counts for 40 MAGs followed by genomic characteristics and taxonomic assignments. The heatmap depicts CAZyme counts (log transformed, normalized, and scaled to a range of −2 to 2) for each category: AA-auxiliary activities, GT-glycosyl transferase, CBM-carbohydrate binding motif, PL-polysaccharide lyase, GH-glycoside hydrolase, and CE-carbohydrate esterase. The grid indicates the presence of specific functional genes, hydrogenase (*hydAB*), methyl coenzyme M reductase (*mcrA*), dissimilatory sulfite reductase (*dsrAB*), formate dependent nitrite reductase (*nrfAH*), periplasmic nitrate reductase complex (*napAB*), dissimilatory nitrate reductase (*narGH*), nitrite reductases (*nirK* and *nirS*), nitric oxide reductase (*norB*), and nitrous oxide reductase (nosZ). The color of each gene column corresponds to the redox processes (*i-vii*) in panel C. The taxonomic assignment for each MAG is designated by the same colors as in panel B. **(B)** Stacked bar-chart representing the microbial community structure based on RPKM values assessed for each recovered bin for each of the three Illumina sequence libraries obtained for each granule, columns A, B, and C. Each bar includes the summed RPKM values for all bins for each assigned taxonomy. “Other” includes bins assigned to low abundance taxa, taxa without a representative high-quality MAG, and all un-binned contigs. **(C)** Diagram of potential biologically mediated redox processes (*i-vii*) occurring within the granules.

The hybridSPAdes assembly of the three short-read (Illumina) and one long-read (Oxford Nanopore) libraries totaled 458,545,694 bases, with an N50 of 15,505 bases, and the longest scaffold at 1,311,200 bases in length – an overall improvement over the short-read (metaSPAdes) assembly (S1 Table in S1 File). An average of 83.8% (± 0.2% SD, n = 3) of the three short-read libraries aligned to the assembly. MaxBin2 binning resulted in 108 bins (S2 Table in S1 File) that were 333,583,683 bases in length (72.7% of the total assembly) and included 68.5% of all assembled scaffolds. Read mapping to the 108 bins and un-binned scaffolds from each short-read library indicated significantly correlated abundance patterns (p < 4e-52, Avg. Pearson’s rho = 0.95 ± 0.02 SD, n = 3) among pairwise comparisons of the three granules. Taxonomic assignment to the 108 bins (S3 Table in S1 File) revealed a diverse microbial community with 18 bins assigned to methanogenic Archaea that accounted for 43% (± 1.1% SD, n = 3) of the metagenomes based on the relative proportion of summed RPKM values. These bins included populations of *Thermoplasmata* (2 bins), *Methanobacteria* (6 bins), and *Methanomicrobia* (10 bins) (Fig 2B). The second and third most abundant taxonomic groups included 13 bins assigned to *Betaproteobacteria* that represented 16% (±3.7% SD, n = 3) of the community, followed by 7 *Desulfobacterota* bins (avg. 8.0% ± 1.2% SD, n = 3). Remaining taxa with greater than 1% relative abundance included *Ca. Aminicenantes* (1 bin, avg. 4.2% ± 1.5% SD), *Bacteroidia* (4 bins, avg. 4.1% ± 0.5% SD), *Anaerolinea* (6 bins, avg. 2.9% ± 0.54% SD), *Krumholzibacteriota* (1 bin, avg. 2.0% ± 1.1% SD), *Ca.* WOR-3 (1 bin, avg. 1.7% ± 0.76% SD), *Gammaproteobacteria* (1 bin, avg. 1.3% ± 1.3% SD), and *Ca. Fermentibacteria* (1 bin, avg. 1.1% ± 0.2% SD) (Fig 2B).

### Functional guilds are phylogenetically cohesive

Of the 108 bins produced by the hybrid assembly, 40 of them (representing an average of 45.7% ± 4.3% of the total assembly RPKM) were of sufficient high quality for further analysis (CheckM completeness ≥ 80% and contamination ≤ 20%) (S2 Table in S1 File) to identify genomic features that differentiate individual taxa and predict their functional roles within the granule consortia. Clustering of these 40 MAGs based on KEGG Orthology (KO) annotations resulted in one singleton cluster plus eight clusters that exhibited significant within group correlation (PERMANOVA, 999 permutations, 9 groups, pseudo-F = 2.48, p ≤ 0.001) and generally contained phylogenetically related taxa within each cluster (Fig 2A). Functional differences between each cluster are highlighted by gene annotations for CAZymes and specific redox genes (Fig 2A). The one singleton cluster (Bin.081 – *Alphaproteobacteria*), was assigned to the genus *Pleomorphomonas*. This MAG had a relatively low abundance (avg. 0.18% ± .05% SD), limited numbers of genes assigned to CAZymes, and no identified genes indicating a role in nitrogen or sulfur respiration. Bin.081 did contain aldehyde and alcohol dehydrogenases, e.g., the fermentative acetaldehyde-alcohol dehydrogenase, *adhE* [27]. *Pleomorphomonas* isolates have exhibited diverse physiologies, including carboxydotrophs, capable of anaerobic carbon monoxide (CO) oxidation [28]. The CO dehydrogenase complex genes *cool* and *cooH* were identified in Bin.081, but not *cooS* and *cooF* [28]. The remaining eight clusters corresponded to the primary functional roles within the granules that include the conventional anaerobic digestion processes (Fig 2C; hydrolysis, acidogenesis, acetogenesis or syntrophic fermentation, and methanogenesis), as well as anaerobic respiration processes (Fig 2C; sulfur transformation, nitrogen transformation, and potentially some roles in iron transformations).

### *Bacteroidia* are the primary hydrolyzers

Cluster 6 included three MAGs assigned to *Bacteroidia* plus two *Planctomycetota* MAGs, Bin.054 assigned to class *Phycisphaerae* and Bin.071 assigned to class *Planctomycetia*. In general, cluster 6 MAGs had the highest content of genes assigned to CAZyme classifications for carbohydrate esterases (CE), glycosyl hydrolases (GH), polysaccharide lyases (PL), and carbohydrate binding motifs (CBM), indicating a principal role in degradation of polymers and thus supplying organic reductant to the biofilm community. The 45 KOs significantly enriched in this cluster (S4 Table in S1 File) support this functional role; these enriched genes included alpha-L-rhamnosidase (*ramA*), beta-galactosidase (*bgaB*, *lacA*), and alpha-L-fucosidase (*fucA*). These findings are in line with prior research indicating that *Bacteroidetes* populations in anaerobic digesters potentially degrade polymers and ferment carbohydrates [29]. Additionally, the taxa represented by the two *Planctomycetota* MAGs have been identified as anaerobic fermenters and genomic analysis of members of the *Phycisphaerae* lineage has indicated a strictly fermentative saccharolytic lifestyle [30,31].

### Acidogenic and acetogenic fermenters formed separate clusters

Five MAGs assigned to *Anaerolinea* comprised cluster 5 (Fig 2A) which had 43 KOs that were significantly enriched (S4 Table in S1 File). Many of these genes were indicative of a general functional role as chemoorganoheterotrophic facultative anaerobic fermenters, using predominantly carbohydrate oligomers as substrates. Among these genes were multiple transporters including ABC multiple sugar transporter, ribose transporter (*rbs*), raffinose, stachyose, melibiose transporter (*msm*), arabinogalactan oligomer/maltooligosaccharide transport system (*cyc*, *gan*, *mdx*), acarbose 7IV-phosphotransferase system (*acb*), nucleoside transporter (*nup*), as well as the *lac*/*gal* regulator system. Although these *Anaerolinea* populations may share a common general niche, deeper analysis of the transporters encoded within each genome suggested finer scale diversity in terms of the types of carbon sources used (Table 1). For example, most of the transporters listed in Table 1 were typically only present in two of the five genomes and specific transporters for glycine, glutamate, N-acetylglucosamine, glycerol, glucitol/sorbitol, and phospholipids were only identified in one of the MAGs. Three of the *Anaerolinea* MAGs harbored the *nrf* gene needed for dissimilatory nitrate reduction to ammonia (DNRA) suggesting some additional roles in nitrogen cycling within the granules.

**Table 1:** Transporter diversity among *Anaerolinea* MAGs.

Transporter	Bin.020	Bin.023	Bin.047	Bin.049	Bin.063
Glycine betaine/proline transport system (Pro)					**X**
General L-amino acid transport system (Aap)	**X**				**X**
Glutamate/aspartate transport system (Glt)			**X**		
N-Acetylglucosamine transport system (ABC.NGC)			**X**		
Multiple sugar transport system (ABC.GGU)	**X**	**X**	**X**		
Putative simple sugar transport system (ABC.SS)			**X**		**X**
Glucose/mannose transport system (Gts)				**X**	**X**
Fructose transport system (Frc)			**X**		**X**
Rhamnose transport system (Rha)			**X**		**X**
D-Xylose transport system (Xyl)		**X**	**X**		
Glucose/mannose transport system (Gts)				**X**	**X**
Raffinose/stachyose/melibiose transport system (Mem)	**X**		**X**	**X**	**X**
Erythritol transport system (Ere)			**X**		
Glycerol transport system (Glp)					**X**
alpha-1,4-Digalacturonate transport system (Agu)	**X**				
alpha-Glucoside transport system (Agl)	**X**		**X**	**X**	**X**
PTS system, galactitol-specific II component (PTS-Gat-EII)		**X**			**X**
PTS system, glucitol/sorbitol-specific II component (PTS-Gut-EII)					**X**
PTS system, ascorbate-specific II component (PTS-Ula-EII)		**X**			
Putative sn-glycerol-phosphate transport system (Ugp)		**X**	**X**		
Phospholipid transport system (Mla, Lin)			**X**		
Putative spermidine/putrescine transport system (ABC.SP)			**X**		**X**

MAGs assigned to four candidate phyla (Bin.077 – *Ca.* FCPU427, Bin.004 – *Ca. Aminicenantes* formerly OP8, Bin.008 *Ca.* WOR-3, and Bin.024 *Ca. Fermentibacteria* formerly Hyd24−12) and one MAG assigned to *Krumholzibacteriota* (Bin.007) formed cluster 8. This cluster had the lowest number of significantly enriched KOs, only 11, likely due to the higher level of phylogenetic diversity within the cluster. All four MAGs were assigned to different phyla and this diversity led to greater within-cluster distances than for the other clusters. Two significantly enriched genes were related to nucleotide sugar synthesis (*rmd* - GDP-4-dehydro-6-deoxy-D-mannose reductase) and polymer transport (*exbB* - biopolymer transport protein) suggesting a role in biofilm formation. Previous research has identified *Ca. Aminicenantes* (OP8) in methane producing environments, such as anaerobic digesters [32] and hydraulic fractured coal beds [33]. *Ca. Aminicenantes* have been linked to cellulose degradation and a putative fermentative saccharolytic lifestyle in deep subsurface aquifers [34,35]. *Ca.* WOR-3 bacteria have been linked to cellulose degradation in estuarine sediments [24]. Analysis of *Ca. Fermentibacteria* genomes suggests a functional role in fermentation and acidogenesis and are commonly reported in anaerobic digesters [36] and anoxic sediments [37]. *Krumholzibacteria* have been identified as slow-growing fermenters [38] with potential roles in iron cycling [39].

Cluster 7 included three MAGs, a *Flavobacteria* (Bin.061) and two *Ignavibacteria* MAGs (Bin.036 and Bin.093) (Fig 2A). Among the 17 KOs identified as enriched in this cluster were a proton dependent oligopeptide transporter and a hemoglobin/transferrin/lactoferrin receptor protein (S4 Table in S1 File). The *Flavobacteria* MAG contained genes annotated as *nirK* and *nirS*. While this might indicate contamination, recent reports have shown both types of *nir* genes can be present in a single organism [40]. The two *Ignavibacteria* MAGs harbored the *nrf* gene for DNRA, consistent with analysis of *Ignavibacteria* MAGs from partial-nitritation anammox reactors [41]. The only *Ignavibacteria* isolate was characterized as an obligately anerobic fermenter [42], but genomic analysis expanded this view identifying nitrite and nitrous oxide reductases, as well as hydrogenase genes that may either generate H_2_ during fermentation or be used to oxidize H_2_ [43].

Cluster 4 consisted of three MAGs assigned to the order *Clostridiales* which are often observed as abundant syntrophic acetogenic fermenters within anaerobic digester systems [44]. Twenty-seven KOs were significantly enriched in this cluster (S4 Table in S1 File), including genes related to sporulation, fatty acid metabolism (fatty acid kinase - *fakB*), lipid metabolism (3-hydroxybutyryl-CoA dehydrogenase -*mmgB*), and fermentation (enoyl-CoA hydratase – *crt*). Two of the MAGs (Bin.056 and Bin.069) were assigned to the family *Syntrophomonadaceae* and possessed one or both subunits for *hydAB* which may function in the proton reduction direction, suggesting these members may drive syntrophic fermentation in association with hydrogenotrophic methanogens.

### The most abundant methanogens were acetoclastic

Cluster 2 included nine MAGs assigned to methanogenic taxa. Of the KOs identified among all these MAGs, 151 were significantly enriched (S4 Table in S1 File). Among these KOs were many archaeal specific genes, including the genes involved in methanogenesis. Among the methanogenic taxa Bin.021 was assigned to *Methanothrix*, of which the first isolate was obtained from an anaerobic digester and characterized as an obligately acetoclastic methanogen [45]. Although Bin.021 was the only high-quality bin representing acetoclastic methanogens in the assembly, two lower quality bins, Bin.001 and Bin.002 assigned to genus *Methanothrix* (S2 Table in S1 File), had the highest average relative abundances (relative to total RPKM) in the three granule libraries, 14.8% ± 1.53% and 7.7% ± 0.89% respectively. The methanogenic taxa also included two MAGs, Bin.045 and Bin.057, assigned to the class *Thermoplasmata* and the genus *Methanomassiliicoccus*, which have been characterized as methane producers that are dependent on H_2_ to reduce methyl compounds [46]. Four MAGs (Bin.055, Bin.018, Bin.005, and Bin.025) in cluster 2 were assigned to the genus *Methanobacterium* within the class *Methanobacteria*. This lineage is generally obligately hydrogenotrophic [47], reducing CO_2_ with H_2_, however, some isolates can utilize formate [48], methanol [49], or secondary alcohols [50]. The remaining three Archaeal MAGs were assigned to class *Methanomicrobia*, with two MAGs (Bin.003 and Bin.048) assigned to the genus *Methanoregula* that can utilize formate [51] or H_2_ for methane production [52].

### *Desulfobacterota* were the primary sulfate reducers

Cluster 3 included 4 MAGs assigned to the phylum *Desulfobacterota*. These MAGs had 53 KOs significantly enriched (S4 Table in S1 File), including genes for sulfite reduction (dissimilatory sulfite reductase – *dsr* and heterodisulfide reductase - *hdrA2*) plus amino acid transporters (*liv* – BCAA transporter and ABC – polar amino acid transporters), genes for anaerobic metabolism (*frd* – fumarate reductase and *acsA* - anaerobic carbon-monoxide dehydrogenase), and the Wood–Ljungdahl pathway (S4 Table in S1 File). All four MAGs also contained hydrogenases (*hydAB*). Three of the *Desulfobacterota* MAGs (Bin.034, Bin.006, and Bin.033) were assigned to the family *Syntrophaceae* which are described as strict anaerobes that may respire (e.g., sulfite reduction) or ferment in syntrophic association with H_2_ oxidizers [53]. These three MAGs also contained the nitrite reductase (*nrf*) utilized in DNRA, indicating a potential role in nitrogen cycling. Bin.059 was assigned to the family *Desulfovibrionaceae*, which like the *Syntrophaceae* are obligate anaerobes, however the *Desulfovibrionaceae* can only oxidize organics to acetate, as opposed to complete oxidation to CO_2_ [54]. Together, these sulfite reducers likely play an important role in the development of the pyrite rich dark layers of the granular biofilm. The reaction of hydrogen sulfide with iron sulfur (FeS + H_2_S ➔ FeS_2_ + H_2_ [55]) releases hydrogen as a substrate for hydrogenotrophic methanogens [56] or potentially other anaerobic hydrogen oxidation reactions, thus providing redox links between sulfate reduction, methanogenesis, and pyrite formation.

### Diverse *Pseudomonadota* comprised the cluster 1 denitrifiers

Cluster 1 (Fig 2A) included one *Gammaproteobacteria* MAG (Bin.106) and four *Betaproteobacteria* MAGs (Bin.015, Bin.026, Bin.012, and Bin.086). All five MAGs contained nitrate and nitrite reductases genes (*nar* and *nir*), and one contained the full denitrification pathway (Bin.015). In total 58 KOs were significantly enriched in this cluster (S4 Table in S1 File), including *cbb*_*3*_-type cytochrome c oxidase, type IV pilus genes, and regulatory genes *fis* and *sspAB*. Bin.106 was assigned to the genus *Thermomonas*, which includes denitrifiers identified in engineered ecosystems [57] and has been isolated from wastewater treatment systems [58,59] and identified in marine sediments [60]. Bin.015 and Bin.026 were both assigned to order *Burkholderiales*. Bin.026 was further assigned to the genus *Diaphorobacter*, which includes several isolates capable of denitrification and utilization of acetate, lactic acid, and other organic acids [61,62]. Bin.086 was assigned to the family *Methylophilaceae* which includes diverse taxa primarily implicated in the oxidation of C1 compounds – methanol and methylated amines [63,64]. Bin.012 was assigned to the family *Thiobacillaceae* and contained dissimilatory sulfite reductase genes (*dsr*), unlike other members of Cluster 1, which may allow nitrate dependent sulfur oxidation. Species within the *Thiobacillaceae* have also been implicated in nitrate-dependent pyrite oxidation [65], and may play an important role in nitrate-dependent iron sulfide dissolution in freshwater ecosystems [66]. Pyrite observed in the dark layers of the granular biofilms [25] may support nitrate-dependent pyrite oxidation (FeS_2_ + 7.5NO_3_^-^ + 3.5H_2_O ➔ Fe(OH)_3_ + 2SO_4_^−2^ + 7.5NO_2_^-^ + 4H^+^) [65] that would regenerate oxidized iron and sulfur compounds that would in turn serve as electron acceptors and lead to regeneration of pyrite in a cryptic redox cycle.

### Comparing granule community to other anaerobic systems

**Fig 3:**
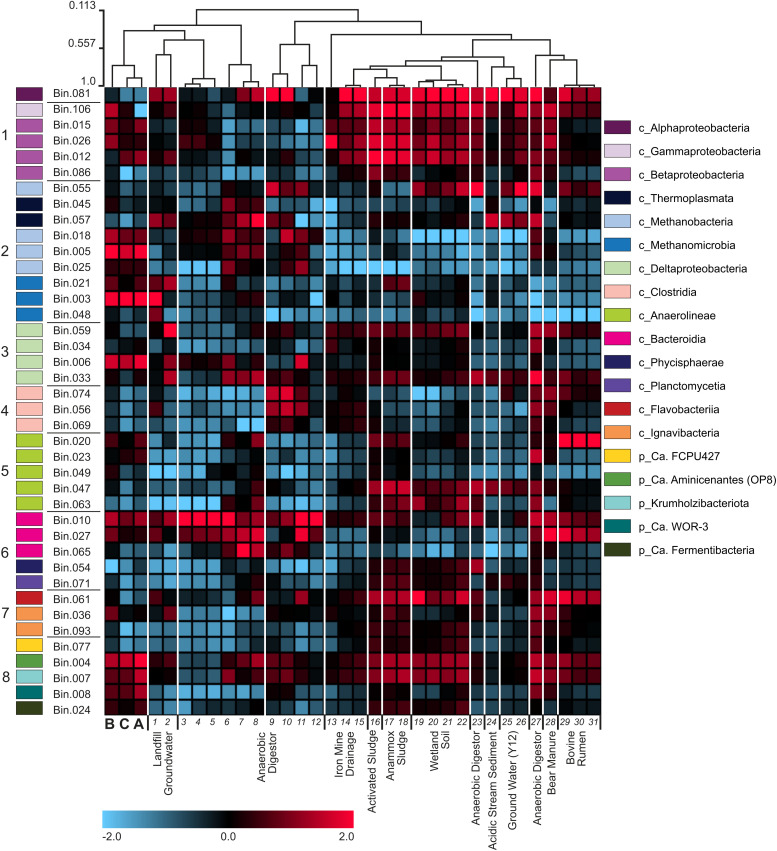
Metagenomic comparisons. Heatmap presenting log normalized and scaled (−2 to 2) RPKM values obtained by mapping reads from 31 metagenomes and the three granule metagenome libraries (**A, B,** and **C** from this study) represented by each column against the 40 high MAGs, each row. Clustering of columns was based on pairwise correlation scores between each sample’s set of bin RPKM values. The order and taxonomy of MAGs in each row is consistent with Fig 1. Heatmap columns for compared metagenomes are numbered and the corresponding descriptions are listed in S5 Table in S1 File.

To place the granule biofilm community into a broader ecological context, we used read recruitment to compare its taxonomic and functional composition to those of diverse anaerobic systems, providing insight into shared and unique microbial features across these environments. Specifically, we assessed how the methanogenic biofilm community that developed under carbon-limited, nitrate-rich conditions compared to communities from other anaerobic digesters, as well as natural and polluted sites likely to harbor microorganisms performing similar anaerobic processes (S5 Table in S1 File). RPKM values for two samples from municipal landfills and six anaerobic digesters were the most correlated with the values observed for the granules in this study (Fig 3 col. *1−12*). The samples that produced the highest observed mapped read percentages (S6 Table in S1 File) included the anaerobic digester samples (Fig 3 col. *3−6*) that correlated well with the three granules in this study, but also included two less correlated samples – the two Y-12 field site groundwater samples (Fig 3 col. *25−26*). The two Y-12 samples had comparatively higher RPKM values for Cluster 1 denitrifiers and reduced RPKM values for Cluster 2 methanogens and Cluster 8 fermenters, suggesting that these samples represent a respiration dependent environment.

The availability of nitrate or nitrite may also explain the higher observed read recruitment by *Beta*- and *Gammaproteobacteria* MAGs (Cluster 1) for the anaerobic granules in this study (Fig 3, col. A, B, and C) compared to other anerobic digester samples (col. *3*–*12*). Samples from other sites that were also likely to support denitrification (activated sludge-col. *16*, anammox reactor sludge-col. *17*–*18*, and wetland soils-col. *19–22*) had higher read recruitment to the Cluster 1 MAGs than other anaerobic digester samples. The opposite trend was observed for the *Bacteroidia* MAGs (Cluster 6) which had higher read recruitment from anaerobic digesters (Fig 3, col. *3–12*) and Bovine rumen (Fig 3, col. *29–31*) samples. This indicates that digester and rumen environments offer a consistent niche space for *Bacteroidia* populations involved in hydrolysis of polymers and fermentation. Read recruitment patterns were less cohesive for other clusters, suggesting that the unique set of selective pressures imposed by distinct environments shape the within guild diversity. These pressures may relate to genomic factors that govern utilization of specific electron donors (e.g., specific types of carbon substrates) and electron acceptors, as well as specific kinetic factors that may influence competitive outcomes under distinct substrate concentrations.

### Conserved functions across different environments

**Fig 4:**
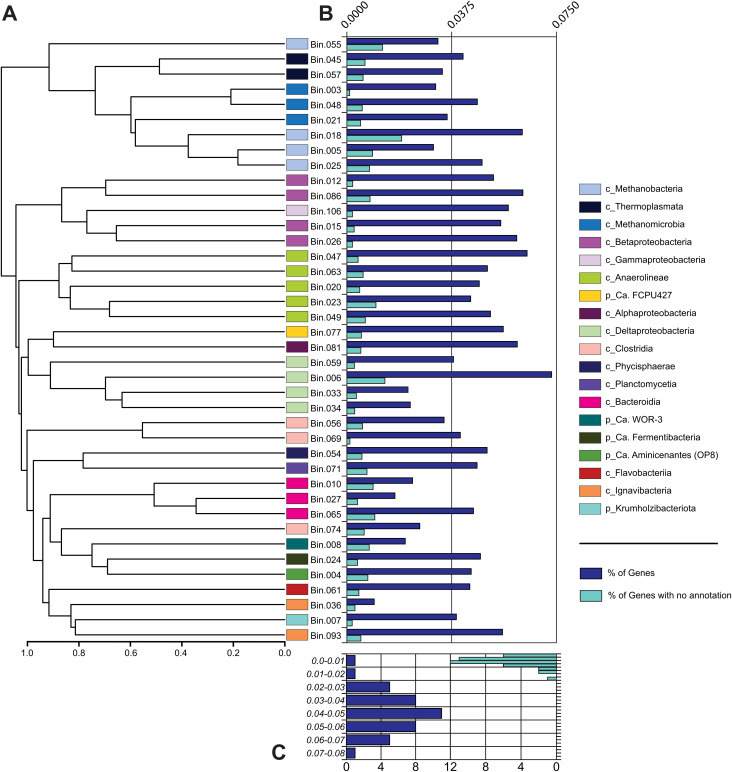
Identification and functional clustering of conserved genes. **(A)** Clustering of 40 MAGs based on the reduced set of KOs identified as conserved among any of the MAGs. Taxonomy is indicated by the legend. **(B)** Percent of genes within each genome identified as having significant read mapping (blue bars) and the percent of genes in each genome identified as significant but not having any annotation assignment from KEGG, eggNOG, or CAZyme database searches (cyan bars). **(C)** The left panel histogram depicts the distribution of MAGs within each range of genome proportion identified as significant (1% width bins, blue bars). The right panel histogram presents the distribution of MAGs based on percent of genes identified as significant but not annotated (0.25% width bins, cyan bars).

Next, we assessed the coverage of individual genes in each MAG across all the compared metagenomes (Fig 3) to identify specific functions that were consistently present in the genomes of closely related organisms. Among the 40 high-quality MAGs, 6,342 genes were identified as conserved (S7 Table in S1 File), i.e., gene sequences that had an average mapped read depth of more than twice the expected value across all metagenome comparisons. Clustering of the 40 MAGs based on this set of conserved genes (Fig 4) resulted in significantly similar pairwise distances (Mantel Test, rho = 0.922, p ≤ 0.001) to the previous analysis that incorporated all KO annotated genes (Fig 2A). PERMANOVA analysis of this reduced gene set distance matrix using the same cluster definitions as in Fig 2 resulted in a higher pseudo-F statistic (999 permutations, 9 groups, pseudo-F = 4.18, p ≤ 0.001) than the previous cluster analysis, indicating a stronger effect of groupings on differences between pairwise distances. There were, however, some minor changes in how the different hydrolytic and fermentative MAGs were clustered. These shifts may indicate more accurate functional guild classifications. The *Clostridia* MAG (Bin.074) clustered more closely with three candidate phyla MAGs (Bin.008, Bin.024, and Bin.004). Of the three *Clostridia* MAGs, Bin.074 was the only one not assigned to the family *Syntrophomonadaceae* and the only one without identified hydrogenase genes (*hydAB*). These characteristics suggest that Bin.074 may have a different fermentation role than the other two *Clostridia*.

On average, 4.3% (±1.5% SD n = 40) of genes in each MAG were identified as conserved, a fraction that was normally distributed across all MAGs (Shapiro-Wilk Test, stat = 0.985, p = 0.858) (Fig 4B). The proportion of these genes that had no KO annotation or COG category assignment averaged 0.59% (±0.38% SD n = 40) and was not normally distributed (Shapiro-Wilk Test, stat = 0.891, p = 0.0011). The annotated conserved genes, often corresponded to genes previously identified as enriched within specific functional guilds including: *cbb*_*3*_ type cytochrome *c*-oxidase in cluster 1, genes required for methanogenesis in cluster 2, amino acid transporters in cluster 3, ribose and nucleoside transporters in cluster 5, glycosyl hydrolases in cluster 6, and a fermentation pathway related gene methylmalonyl-CoA mutase [67] in cluster 7. While this analysis does not indicate that the specific genomes recovered from the granules sequenced in this study are representative of the same species found in the compared environmental samples, it does suggest that the identified genes are conserved among closely related organisms that may perform similar functions in those environments.

## Discussion

Catalytic biomass within anaerobic digesters converts organic material through a set of sequential processes (i.e., hydrolysis, acidogenesis, acetogenesis and methanogenesis) carried out by different microbial functional guilds. In these systems, overall performance is governed by a number of reactor-specific environmental and operational parameters [68]. The addition of iron to anaerobic digesters is a common operation performed to enhance organic matter removal and reduce odors associated with hydrogen sulfide (H_2_S) production [69] and also provides an important trace mineral required for enzymes used in methanogenesis [70]. The granules analyzed in this study were based on our previous research that revealed a unique biofilm morphology of alternating light biomass-rich and dark FeS/FeS_2_-rich layers. This morphology developed in an anaerobic digester with repeated iron additions, followed by exposure to nitrate-rich conditions in dedicated lab reactor studies aimed at nitrate removal without addition of any organic carbon [25]. Nitrate reduction was linked to the cryptic oxidation of reduced iron and sulfur compounds as well as organic carbon from within the granule [25]. In addition to the research goal of assessing the feasibility of utilizing anaerobic digester granules for treatment of nitrate polluted waters [25], we sought to examine the impacts of nitrate addition in terms of microbial populations enriched following the change in redox conditions and further compare the resulting community to other natural and engineered ecosystems.

### Defining microbial community structure in terms of functional guilds

We compared the functional content of 40 high-quality genome bins recovered from the assembly and identified significantly distinct clusters that corresponded to functional guilds with the genomic potential to perform metabolic roles expected to occur within the system (Fig 2). This approach revealed taxonomic cohesiveness among the organisms within each of these broad functional groups. Within each of these functional groups we observed finer scale niche adaptations, such as the different sugar transporter repertoires observed for the *Anaerolinea* MAGs (Table 1). This implies that functional units within a microbial community are operationally defined and reflect both the specific ecosystem and the research focus. For this study we were primarily concerned with identifying the populations responsible for the general metabolic roles (i.e., hydrolysis, fermentation, methanogenesis, nitrate reduction, and sulfate reduction) to describe the observed system level functions, i.e., nitrate reduction without addition of exogenous reductant. Activity-based batch tests in our earlier research [25] were supported by the functional classification of recovered MAGs presented here, which also demonstrated that the applied selective pressure promoted the growth of a community enriched in functional genes supporting N, S, Fe and C cycling in the bioreactor system. Future metagenomics analyses should leverage genomic clustering approaches to develop quantitative metrics for functional characterization of microbial communities that build upon macro ecological concepts such as functional diversity or functional composition [71,72]. Microbial functional diversity metrics would facilitate comparisons of within community (i.e., alpha diversity) or between community (i.e., beta diversity) that could elucidate community assembly rules and identify relationships between phylogenetic and functional diversity.

### Environmental relevance of a redox-enriched anaerobic granule community

Metagenomic analysis revealed some similarities between the granular biofilms in this study and prior investigations of anaerobic digester communities [14,21,29], with shared diverse taxa responsible for organic matter hydrolysis, acidogenesis, and syntrophic acetogenesis as well as abundant methanogenic populations. These similarities were also observed through direct comparisons with anaerobic digester metagenomes (Fig 3). However, the supply of nitrate and sulfate enriched the community in populations capable of anaerobic respiration and potentially capable of reducing iron or involved in the oxidation of reduced sulfate or iron (including iron sulfur minerals such as pyrite). Pyrite oxidation plays an important role in natural environments [73], serving as a link between iron, sulfur, and nitrogen cycles in marine sediments [26,74] and wetlands [66]. Furthermore, the utilization of pyrite to enhance denitrification in engineered ecosystems has also been explored with some success [25,75–77]. Although not included in the analysis of high-quality MAGs, one of the lower quality genome bins was associated with taxa that have been implicated in pyrite oxidation. *Thiobacillus* (Bin.108) has been reported to couple denitrification with the oxidation of iron sulfides [78,79]. While the role of iron and iron sulfur redox metabolisms was likely important to the biofilm community examined in this study, characterizing the functions of iron oxidizing and reducing taxa within metagenomes remains challenging as annotation of iron redox genes requires contiguous assemblies that enable characterization of operon structures [80]. The development of less phylogenetically complex and more experimentally tractable model systems that mimic natural microbial communities will enable more mechanistic insight into the impacts of environmental changes [81,82] such as the impact of nitrate pollution on groundwater ecosystems or the impact of sulfate intrusion on wetland soils following climate change induced sea level rise [83,84].

**Fig 5:**
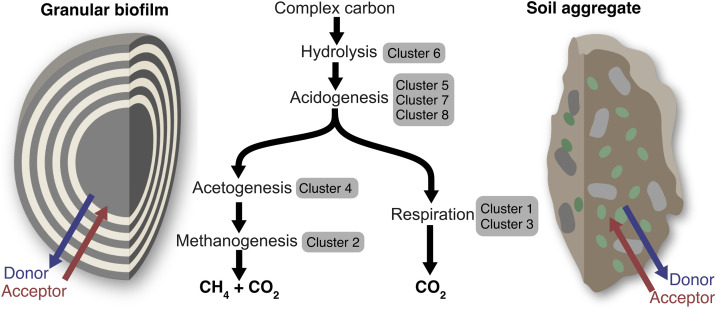
Granular biofilms as model systems for complex soil ecosystems. The granular biofilms examined in this study possess microbial functional guilds, clusters 1-8, that perform similar functions as in the more complex microbial communities of natural soil aggregates.

The anaerobic granular biofilms in this study represent such a model system, containing functional guilds involved in hydrolysis, fermentation, methanogenesis, and anaerobic respiration that link carbon, sulfur, nitrogen, and iron cycles (Fig 5). Furthermore, specific genes related to these diverse functional roles were identified in microbial populations found in natural and engineered ecosystems (Fig 4). In addition to containing representative populations possessing diverse metabolic functionality found in natural anoxic ecosystems, the structure of granular biofilms provides similar chemical gradients. The granular biofilm microbial community functions within a counter-diffusive environment: hydrolysis and fermentation products diffuse outward, while nitrate and sulfate supplied in the media diffuse inward. This shapes chemical gradients and transitory niche spaces along which competition for substrates would likely result in a gradual shift from methanogens to sulfate- and nitrate-reducers. The community conserves energy by linking carbon and hydrogen oxidation with sulfur, iron, and nitrogen redox cycles. This suggests that the observed differences in community structure compared to anaerobic digesters, e.g., the abundance of facultative anaerobic denitrifiers, and the observed nitrate removal is dependent upon the availability of organic compounds in the outer layers of the biofilm where inward diffusing nitrate is not yet depleted. This counter-diffusive gradient of organics and nitrate would provide a range of nitrate to organic carbon ratios, potentially favoring denitrification at the granule exterior and DNRA toward the carbon rich and nitrate depleted granule interior [85].

### Study implications and limitations

Using a genome-centric approach, this study demonstrated how clustering MAGs by their functional gene composition can reveal functional guilds within anaerobic granules. This strategy has broader applications for identifying guilds that drive nutrient transformations in other ecosystems, including those with uncultured microorganisms lacking physiological data. Our comparative analysis also showed that these functional guilds are relevant across diverse anaerobic environments.

Although we analyzed only three individual granules, their consistent taxonomic profiles suggest they were representative of the broader bioreactor community, and the metagenome comparisons supported the relevance of our findings beyond this specific system. While this approach is based on functional potential rather than direct in situ activity, the correspondence between identified guild members and known cultured representatives indicates that the results remain biologically significant.

## Conclusion

By investigating a relatively low complexity microbial community, we were able to characterize the abundant taxa according to their functional roles, highlighting the utility of engineered ecosystems as model systems for microbial ecology. Taxa were differentiated into functional guilds based on their annotated genomic features and incorporated publicly available metagenomic data to better characterize the microbial community and further delineate the functional roles of specific taxa based on biogeographic distribution patterns and gene specific selective pressures. While natural environments are often too complex to manipulate experimentally, engineered systems offer controlled conditions and tractable diversity, enabling targeted investigation of microbial community assembly and responses to perturbations, such as nitrate addition in this study. The granules in this study demonstrated how enrichment of nitrate and sulfate reducers from an anaerobic digester community can lead to a functionally diverse microbial community, however, similar systems could be used to investigate the structural and functional changes from anthropogenic impacts on naturally occurring low redox potential environments.

## Materials and methods

### Origin of granular biofilms

The origin of the granular biofilms was described previously [25]. Briefly, anaerobic granules from a full-scale upflow anaerobic sludge blanket reactor fed with organic carbon, ferric iron, and sulfate were used to seed a 40 L pilot-scale rotating drum reactor and 3.5 L lab-scale sequencing batch reactor. Both reactor systems were fed anoxically with nitrate and no organic carbon at either 20^o^C or 30^o^C depending on the phase. These reactors demonstrated the capacity for nitrate removal connected to iron, sulfur, and organic carbon compounds contained within the granules. Nitrate removal rates ranged from 0.25–4.83 mgNO_3_^-^•gVSS^-1^•d^-1^ depending on phase.

### Metagenome sequencing, assembly, and binning

Three individual granules from the pilot-scale reactor exhibiting layered biofilm structure (Fig 1) were selected for separate DNA extractions using the Qiagen DNEasy Power Biofilm Kit (Qiagen, Germany). Individual granules were used instead of pooling biomass to capture potential inter-granule variability. DNA was quantified using a NanoDrop 2000 (Thermo Fisher Scientific, Wilmington, Germany). DNA aliquots from each granule were sent to BGI America for library preparation and sequencing on the Illumina NovaSeq platform producing 2x150 bp paired end reads. The three libraries (A, B, and C) contained 49,050,464, 49,117,576, and 48,706,712 paired end reads respectively. Reads were uploaded to the DOE KBase platform [86], merged into one library with 146,964,752 reads, then quality filtered and trimmed using Trimmomatic [87] resulting in a single library with 145,235,876 paired reads. An aliquot of DNA from one granule (granule C) was selected for long-read sequencing using the Oxford Nanopore MinION [88] platform. The Rapid Sequencing Kit (SQK-RAD004) was used for library preparation and run on a single flow-cell. Base calling was performed locally using Albacore (Oxford Nanopore Technologies) and reads shorter than 1,000 bases were removed, resulting in 1,053,672 reads totaling 3,866,526,020 bases and a mean length of 3,669.57 bases. MinION reads were uploaded to DOE KBase [86] and assembled along with the combined Illumina short read library using hybridSPAdes [89]. The combined Illumina libraries were also assembled in parallel with metaSPADEs [90] using the default parameters and was compared with the hybridSPAdes [89] assembly using QUAST [91] (S1 Table in S1 File). Both assemblies (hybrid and short-read) were binned using MaxBin2 [92] and assessed with CheckM [93] (S2 Table in S1 File). Illumina libraries for the three individual granules were mapped against the assemblies with Bowtie2 [94] to determine how much of each sequence library was represented by each assembly (S1 Table in S1 File) and assess the relative abundance of binned contigs as Reads Per Kilobase of transcript per Million mapped reads (RPKM) values (S6 Table in S1 File). The hybrid assembly was longer, more contiguous, captured more of the sequence libraries, and had less ambiguities (S1 Table in S1 File) so it was selected for further analysis. Taxonomy for bins recovered from the hybrid assembly was assigned using GTDB-Tk v1.6.0 [95], manually checked with phylogenetic trees using the KBase [86] SpeciesTreeBuilder app [96], and converted to National Center for Biotechnology Information (NCBI) nomenclature using the GTDB website (gtdb.ecogenomic.org) (S3 Table in S1 File).

### Functional annotation and analysis

Gene calling for the hybrid assembly was performed using PRODIGAL [97]. Functional annotation of predicted coding sequences (CDS) for the entire assembly was performed with several tools. EggNOGmapper.py [98] was used to search against the EggNOG [99] database in diamond [100] mode. Carbohydrate active enzymes (CAZyme) were annotated using diamond [100] searches against the dbCAN database [101]. Kegg Orthology (KO) assignments were performed with GhostKOALA [102] (https://www.kegg.jp/ghostkoala/). FeGenie [80] was used to search for iron redox genes. High quality bins, those with ≥ 80% completeness and ≤ 20% contamination as assessed by CheckM [93] (S2 Table in S1 File) were also annotated using the DRAM [103] pipeline as implemented in KBase [86].

Forty high-quality bins (i.e., MAGs) were clustered based on KO content using tools from the SciPy spatial.distance and cluster.hierarchy libraries. Prior to clustering, MAG KO counts were normalized by subtracting the average relative abundance of KOs in all MAGs from the observed relative abundance of KOs in each individual MAG and dividing by the average relative abundance of KOs in all MAGs. In other words, the normalized KO array for MAG[i] would be calculated as shown in Eq. 1.


$$Normalized\;KO\;array_{MAG[i]}=\frac{\left(KO\_rel\_abun_{MAG[i]}-mean(KO\_rel\_ab{un}_{All\;MAGs})\right)}{mean(KO\_rel\_ab{un}_{All\;MAGs})}$$ (1)

Functionally similar clusters of MAGs were manually identified from the dendrogram that was created using python scripts and the SciPy pdist (using Pearson correlation scores), SciPy linkage, and SciPy dendrogram libraries. PERMANOVA and Mantel tests were performed using scikit-bio (scikit-bio.org). Non-parametric tests for identifying significantly enriched KO assignments within functional clusters were performed by comparing observed counts for each KO identified among all members of each cluster to KO count distributions generated from 10,000 random samples of annotations of equivalent genome sizes (S4 Table in S1 File). Clustering based on a reduced set of conserved KOs utilized the same methods as clustering with the entire KO set.

### Metagenome comparisons

The 40 high-quality MAGs were compared to 32 publicly available metagenomes downloaded from NCBI SRA (S5 Table in S1 File). The metagenomes were selected to represent diverse anaerobic environments and were required to have been sequenced using Illumina short-read technology with a sufficient depth (> 2 million reads). Sequences were mapped to the hybrid assembly using Bowtie2, and RPKM values for MAGs were calculated (S6 Table in S1 File). MAGs were clustered based on normalized RPKM values across all mapped sequence libraries and distinct clusters were compared based on taxonomic and functional content. The biogeographic distribution of individual gene sequences within MAGs was examined to identify the functional distribution of highly conserved gene sequences. Sequence alignments were converted to mpileup format [104]. Then position specific coverage information was used to determine the coverage for each gene within each of the 40 high-quality MAGs across all 32 compared metagenomes. Individual genes were identified as conserved if coverage from mapped reads was substantially higher than the null expectation that reads would align evenly across the MAG. Specifically they were identified as conserved if the log base 2 of the average percent difference (*D*) between the observed (*o*) and the expected (*e*) under the null condition was greater than 0 for *n* different metagenome libraries (i.e., the average observed coverage was at least two times the average expected coverage)(Eq. 2):


$$\begin{array}{c} D=\frac{\sum_{i=1}^{n}\frac{o_{i}-e_{i}}{e_{i}}}{n} \end{array}$$ (2)

Identified conserved genes for the 40 MAGs are listed in S7 Table in S1 File. Clustering of MAGs based on KO assignments limited to the set of genes identified as conserved was previously described in the previous section.

## Supporting information

S1 File **S1 Table. Sequencing, assembly, and binning statistics. S2 Table. Bin quality and coverage statistics. S3 Table. Taxonomic assignment of bins. S4 Table. Significant genes for functional clusters. S5 Table. Publicly available metagenomic libraries used for comparisons.** SRA accension numbers, sample names, study names, number of reads, and alignment rate is detailed. **S6 Table. Bin coverage based on read-mapping.** Reads per kilobase per million mapped reads (RPKM) values from mapping reads from metagenome libraries, including publicly available ones, to the set of bins. **S7 Table. Conserved genes in MAGs.** Genes identified as conserved in MAGs based on metagenome comparison.
(ZIP)

## References

[1] McHugh S, O’Reilly C, Mahony T, Colleran E, O’Flaherty V. Anaerobic Granular Sludge Bioreactor Technology. Rev Environ Sci Technol. 2003;2:225–45.

[2] Skiadas IV, Gavala HN, Schmidt JE, Ahring BK. Anaerobic Granular Sludge and Biofilm Reactors. In: Ahring BK, Ahring BK, Angelidaki I, Dolfing J, EUegaard L, Gavala HN, editors. Biomethanation II. Berlin, Heidelberg: Springer Berlin Heidelberg. 2003. p. 35–67. 10.1007/3-540-45838-7_2 12747565

[3] O’Flaherty V, Collins G, Mahony T. The Microbiology and Biochemistry of Anaerobic Bioreactors with Relevance to Domestic Sewage Treatment. Rev Environ Sci Biotechnol. 2006;5(1):39–55. doi: 10.1007/s11157-005-5478-8

[4] Nicolella C, van Loosdrecht MC, Heijnen JJ. Wastewater treatment with particulate biofilm reactors. J Biotechnol. 2000;80(1):1–33. doi: 10.1016/s0168-1656(00)00229-7 10862983

[5] Winkler MKH, Kleerebezem R, Kuenen JG, Yang J, van Loosdrecht MCM. Segregation of biomass in cyclic anaerobic/aerobic granular sludge allows the enrichment of anaerobic ammonium oxidizing bacteria at low temperatures. Environ Sci Technol. 2011;45(17):7330–7. doi: 10.1021/es201388t 21744798

[6] Winkler M-KH, van Loosdrecht MCM. Intensifying existing urban wastewater. Science. 2022;375(6579):377–8. doi: 10.1126/science.abm3900 35084964

[7] Wolin MJ, Miller TL, Stewart CS. Microbe-microbe interactions. The Rumen Microbial Ecosystem. Dordrecht: Springer Netherlands. 1997. p. 467–91. doi: 10.1007/978-94-009-1453-7_11

[8] Yarwood SA. The role of wetland microorganisms in plant-litter decomposition and soil organic matter formation: a critical review. FEMS Microbiol Ecol. 2018;94(11):10.1093/femsec/fiy175. doi: 10.1093/femsec/fiy175 30169564

[9] Poh PE, Gouwanda D, Mohan Y, Gopalai AA, Tan HM. Optimization of wastewater anaerobic digestion using mechanistic and meta-heuristic methods: current limitations and future opportunities. Water Conserv Sci Eng. 2016;1(1):1–20. doi: 10.1007/s41101-016-0001-3

[10] M. V. Thomas, R. A. Nordstedt. Generic anaerobic digestion model for the simulation of various reactor types and substrates. Transactions of the ASAE. 1993;36(2):537–44. doi: 10.13031/2013.28370

[11] Husain A. Mathematical models of the kinetics of anaerobic digestion—a selected review. Biomass and Bioenergy. 1998;14(5–6):561–71. doi: 10.1016/s0961-9534(97)10047-2

[12] Batstone DJ, Keller J, Angelidaki I, Kalyuzhnyi SV, Pavlostathis SG, Rozzi A. The IWA Anaerobic Digestion Model No 1 (ADM1). Water Sci Technol. 2002;45:65–73. 12188579

[13] Vanwonterghem I, Jensen PD, Ho DP, Batstone DJ, Tyson GW. Linking microbial community structure, interactions and function in anaerobic digesters using new molecular techniques. Curr Opin Biotechnol. 2014;27:55–64. doi: 10.1016/j.copbio.2013.11.004 24863897

[14] Treu L, Kougias PG, Campanaro S, Bassani I, Angelidaki I. Deeper insight into the structure of the anaerobic digestion microbial community; the biogas microbiome database is expanded with 157 new genomes. Bioresour Technol. 2016;216:260–6. doi: 10.1016/j.biortech.2016.05.081 27243603

[15] Vanwonterghem I, Jensen PD, Rabaey K, Tyson GW. Genome-centric resolution of microbial diversity, metabolism and interactions in anaerobic digestion. Environ Microbiol. 2016;18(9):3144–58. doi: 10.1111/1462-2920.13382 27317862

[16] Campanaro S, Treu L, Rodriguez-R LM, Kovalovszki A, Ziels RM, Maus I, et al. New insights from the biogas microbiome by comprehensive genome-resolved metagenomics of nearly 1600 species originating from multiple anaerobic digesters. Biotechnol Biofuels. 2020;13:25. doi: 10.1186/s13068-020-01679-y 32123542 PMC7038595

[17] Owusu-Agyeman I, Eyice Ö, Cetecioglu Z, Plaza E. The study of structure of anaerobic granules and methane producing pathways of pilot-scale UASB reactors treating municipal wastewater under sub-mesophilic conditions. Bioresour Technol. 2019;290:121733. doi: 10.1016/j.biortech.2019.121733 31301569

[18] Trego AC, Galvin E, Sweeney C, Dunning S, Murphy C, Mills S. Growth and break-up of methanogenic granules suggests mechanisms for biofilm and community development. Front Microbiol. 2020;11:1126. 32582085 10.3389/fmicb.2020.01126 PMC7285868

[19] Kougias PG, Treu L, Campanaro S, Zhu X, Angelidaki I. Dynamic functional characterization and phylogenetic changes due to Long Chain Fatty Acids pulses in biogas reactors. Sci Rep. 2016;6:28810. doi: 10.1038/srep28810 27353502 PMC4926282

[20] Zhang W, Werner JJ, Agler MT, Angenent LT. Substrate type drives variation in reactor microbiomes of anaerobic digesters. Bioresour Technol. 2014;151:397–401. 24183494 10.1016/j.biortech.2013.10.004

[21] Steinberg LM, Regan JM. Phylogenetic comparison of the methanogenic communities from an acidic, oligotrophic fen and an anaerobic digester treating municipal wastewater sludge. Appl Environ Microbiol. 2008;74(21):6663–71. doi: 10.1128/AEM.00553-08 18776026 PMC2576706

[22] Mutschlechner M, Praeg N, Illmer P. Soil-Derived Inocula Enhance Methane Production and Counteract Common Process Failures During Anaerobic Digestion. Front Microbiol. 2020;11: 572759. 33193175 10.3389/fmicb.2020.572759 PMC7606279

[23] Rillig MC, Muller LA, Lehmann A. Soil aggregates as massively concurrent evolutionary incubators. ISME J. 2017;11(9):1943–8. doi: 10.1038/ismej.2017.56 28409772 PMC5563948

[24] Baker BJ, Lazar CS, Teske AP, Dick GJ. Genomic resolution of linkages in carbon, nitrogen, and sulfur cycling among widespread estuary sediment bacteria. Microbiome. 2015;3:14. doi: 10.1186/s40168-015-0077-6 25922666 PMC4411801

[25] Pelivano B, Bryson S, Hunt KA, Denecke M, Stahl DA, Winkler M. Application of pyritic sludge with an anaerobic granule consortium for nitrate removal in low carbon systems. Water Res. 2022;209:117933. doi: 10.1016/j.watres.2021.117933 34923445

[26] Holmkvist L, Ferdelman TG, Jørgensen BB. A cryptic sulfur cycle driven by iron in the methane zone of marine sediment (Aarhus Bay, Denmark). Geochimica et Cosmochimica Acta. 2011;75(12):3581–99. doi: 10.1016/j.gca.2011.03.033

[27] Kim G, Azmi L, Jang S, Jung T, Hebert H, Roe AJ, et al. Aldehyde-alcohol dehydrogenase forms a high-order spirosome architecture critical for its activity. Nat Commun. 2019;10(1):4527. doi: 10.1038/s41467-019-12427-8 31586059 PMC6778083

[28] Esquivel-Elizondo S, Maldonado J, Krajmalnik-Brown R. Anaerobic carbon monoxide metabolism by Pleomorphomonas carboxyditropha sp. nov., a new mesophilic hydrogenogenic carboxydotroph. FEMS Microbiol Ecol. 2018;94(6):10.1093/femsec/fiy056. doi: 10.1093/femsec/fiy056 29741624

[29] Campanaro S, Treu L, Kougias PG, Luo G, Angelidaki I. Metagenomic binning reveals the functional roles of core abundant microorganisms in twelve full-scale biogas plants. Water Res. 2018;140:123–34. doi: 10.1016/j.watres.2018.04.043 29704757

[30] Spring S, Bunk B, Spröer C, Rohde M, Klenk H-P. Genome biology of a novel lineage of planctomycetes widespread in anoxic aquatic environments. Environ Microbiol. 2018;20(7):2438–55. doi: 10.1111/1462-2920.14253 29697183

[31] Dedysh SN, Kulichevskaya IS, Beletsky AV, Ivanova AA, Rijpstra WIC, Damsté JSS. Lacipirellula parvula gen. nov., sp. nov., representing a lineage of planctomycetes widespread in low-oxygen habitats, description of the family Lacipirellulaceae fam. nov. and proposal of the orders Pirellulales ord. nov., Gemmatales ord. nov. and Isosphaerales ord. nov. Syst Appl Microbiol. 2020;43:126050. 31882205 10.1016/j.syapm.2019.126050 PMC6995999

[32] Kuroda K, Nobu MK, Mei R, Narihiro T, Bocher BTW, Yamaguchi T, et al. A Single-Granule-Level Approach Reveals Ecological Heterogeneity in an Upflow Anaerobic Sludge Blanket Reactor. PLoS One. 2016;11(12):e0167788. doi: 10.1371/journal.pone.0167788 27936088 PMC5147981

[33] Robbins SJ, Evans PN, Parks DH, Golding SD, Tyson GW. Genome-Centric Analysis of Microbial Populations Enriched by Hydraulic Fracture Fluid Additives in a Coal Bed Methane Production Well. Front Microbiol. 2016;7:731. doi: 10.3389/fmicb.2016.00731 27375557 PMC4897734

[34] Farag IF, Davis JP, Youssef NH, Elshahed MS. Global patterns of abundance, diversity and community structure of the Aminicenantes (candidate phylum OP8). PLoS One. 2014;9(3):e92139. doi: 10.1371/journal.pone.0092139 24637619 PMC3956909

[35] Kadnikov VV, Mardanov AV, Beletsky AV, Karnachuk OV, Ravin NV. Genome of the candidate phylum Aminicenantes bacterium from a deep subsurface thermal aquifer revealed its fermentative saccharolytic lifestyle. Extremophiles. 2019;23(2):189–200. doi: 10.1007/s00792-018-01073-5 30600356

[36] Kirkegaard RH, Dueholm MS, McIlroy SJ, Nierychlo M, Karst SM, Albertsen M, et al. Genomic insights into members of the candidate phylum Hyd24-12 common in mesophilic anaerobic digesters. ISME J. 2016;10(10):2352–64. doi: 10.1038/ismej.2016.43 27058503 PMC5030696

[37] Saad S, Bhatnagar S, Tegetmeyer HE, Geelhoed JS, Strous M, Ruff SE. Transient exposure to oxygen or nitrate reveals ecophysiology of fermentative and sulfate-reducing benthic microbial populations. Environ Microbiol. 2017;19:4866–81. 28836729 10.1111/1462-2920.13895 PMC5763382

[38] Youssef NH, Farag IF, Hahn CR, Premathilake H, Fry E, Hart M, et al. Candidatus Krumholzibacterium zodletonense gen. nov., sp nov, the first representative of the candidate phylum Krumholzibacteriota phyl. nov. recovered from an anoxic sulfidic spring using genome resolved metagenomics. Syst Appl Microbiol. 2019;42(1):85–93. doi: 10.1016/j.syapm.2018.11.002 30477901

[39] Dalcin Martins P, de Jong A, Lenstra WK, van Helmond NAGM, Slomp CP, Jetten MSM, et al. Enrichment of novel Verrucomicrobia, Bacteroidetes, and Krumholzibacteria in an oxygen-limited methane- and iron-fed bioreactor inoculated with Bothnian Sea sediments. Microbiologyopen. 2021;10(1):e1175. doi: 10.1002/mbo3.1175 33650794 PMC7914226

[40] Sánchez C, Minamisawa K. Redundant roles of Bradyrhizobium oligotrophicum Cu-type (NirK) and cd1-type (NirS) nitrite reductase genes under denitrifying conditions. FEMS Microbiol Lett. 2018;365(5):10.1093/femsle/fny015. doi: 10.1093/femsle/fny015 29361081

[41] Bryson SJ, Hunt KA, Stahl DA, Winkler M-KH. Metagenomic Insights Into Competition Between Denitrification and Dissimilatory Nitrate Reduction to Ammonia Within One-Stage and Two-Stage Partial-Nitritation Anammox Bioreactor Configurations. Front Microbiol. 2022;13:825104. doi: 10.3389/fmicb.2022.825104 35547121 PMC9083452

[42] Iino T, Mori K, Uchino Y, Nakagawa T, Harayama S, Suzuki KI. Ignavibacterium album gen. nov., sp. nov., a moderately thermophilic anaerobic bacterium isolated from microbial mats at a terrestrial hot spring and proposal of Ignavibacteria classis nov., for a novel lineage at the periphery of green sulfur bacteria. Int J Syst Evol Microbiol. 2010;60:1376–82. 19671715 10.1099/ijs.0.012484-0

[43] Liu Z, Frigaard N-U, Vogl K, Iino T, Ohkuma M, Overmann J, et al. Complete Genome of Ignavibacterium album, a Metabolically Versatile, Flagellated, Facultative Anaerobe from the Phylum Chlorobi. Front Microbiol. 2012;3:185. doi: 10.3389/fmicb.2012.00185 22661972 PMC3362086

[44] Cai M, Wilkins D, Chen J, Ng S-K, Lu H, Jia Y, et al. Metagenomic Reconstruction of Key Anaerobic Digestion Pathways in Municipal Sludge and Industrial Wastewater Biogas-Producing Systems. Front Microbiol. 2016;7:778. doi: 10.3389/fmicb.2016.00778 27252693 PMC4879347

[45] Huser BA, Wuhrmann K, Zehnder AJB. Methanothrix soehngenii gen. nov. sp. nov., a new acetotrophic non-hydrogen-oxidizing methane bacterium. Arch Microbiol. 1982;132:1–9. 10.1007/BF00407022 6769415

[46] Borrel G, Parisot N, Harris HMB, Peyretaillade E, Gaci N, Tottey W, et al. Comparative genomics highlights the unique biology of Methanomassiliicoccales, a Thermoplasmatales-related seventh order of methanogenic archaea that encodes pyrrolysine. BMC Genomics. 2014;15:679. doi: 10.1186/1471-2164-15-679 25124552 PMC4153887

[47] Boone DR, Mah RA. Genus I. Methanobacterium. Bergey’s Manual of Systematic Bacteriology. 2001. p. 215–8.

[48] Schauer NL, Ferry JG. Metabolism of formate in Methanobacterium formicicum. J Bacteriol. 1980;142(3):800–7. doi: 10.1128/jb.142.3.800-807.1980 6769911 PMC294100

[49] Borrel G, Joblin K, Guedon A, Colombet J, Tardy V, Lehours A-C, et al. Methanobacterium lacus sp. nov., isolated from the profundal sediment of a freshwater meromictic lake. Int J Syst Evol Microbiol. 2012;62(Pt 7):1625–9. doi: 10.1099/ijs.0.034538-0 21890730

[50] Cadillo-Quiroz H, Bräuer SL, Goodson N, Yavitt JB, Zinder SH. Methanobacterium paludis sp. nov. and a novel strain of Methanobacterium lacus isolated from northern peatlands. Int J Syst Evol Microbiol. 2014;64(Pt 5):1473–80. doi: 10.1099/ijs.0.059964-0 24449792

[51] Yashiro Y, Sakai S, Ehara M, Miyazaki M, Yamaguchi T, Imachi H. Methanoregula formicica sp. nov., a methane-producing archaeon isolated from methanogenic sludge. Int J Syst Evol Microbiol. 2011;61:53–9. 19667393 10.1099/ijs.0.014811-0

[52] Bräuer SL, Cadillo-Quiroz H, Ward RJ, Yavitt JB, Zinder SH. Methanoregula boonei gen. nov., sp. nov., an acidiphilic methanogen isolated from an acidic peat bog. Int J Syst Evol Microbiol. 2011;61:45–52. 20154331 10.1099/ijs.0.021782-0

[53] Kuever J. The Family Syntrophaceae. In: Rosenberg E, DeLong EF, Lory S, Stackebrandt E, Thompson F, editors. The Prokaryotes: Deltaproteobacteria and Epsilonproteobacteria. Berlin, Heidelberg: Springer Berlin Heidelberg. 2014. p. 281–8.

[54] Kuever J. The family Desulfovibrionaceae. In: Rosenberg E, DeLong EF, Lory S, Stackebrandt E, Thompson F, editors. The Prokaryotes: Deltaproteobacteria and Epsilonproteobacteria. Berlin, Heidelberg: Springer Berlin Heidelberg. 2014. p. 107–33.

[55] Rickard D, Luther GW 3 rd. Chemistry of iron sulfides. Chem Rev. 2007;107:514–62. 17261073 10.1021/cr0503658

[56] Thiel J, Byrne JM, Kappler A, Schink B, Pester M. Pyrite formation from FeS and H2S is mediated through microbial redox activity. Proc Natl Acad Sci U S A. 2019;116(14):6897–902. doi: 10.1073/pnas.1814412116 30886102 PMC6452648

[57] McIlroy SJ, Starnawska A, Starnawski P, Saunders AM, Nierychlo M, Nielsen PH, et al. Identification of active denitrifiers in full-scale nutrient removal wastewater treatment systems. Environ Microbiol. 2016;18(1):50–64. doi: 10.1111/1462-2920.12614 25181571

[58] Mergaert J, Cnockaert MC, Swings J. Thermomonas fusca sp. nov. and Thermomonas brevis sp. nov., two mesophilic species isolated from a denitrification reactor with poly(epsilon-caprolactone) plastic granules as fixed bed, and emended description of the genus Thermomonas. Int J Syst Evol Microbiol. 2003;53(Pt 6):1961–6. doi: 10.1099/ijs.0.02684-0 14657130

[59] Ju J-H, Kim J-S, Lee D-H, Jeon JH, Heo S-Y, Seo J-W, et al. Thermomonas aquatica sp. nov., isolated from an industrial wastewater treatment plant. Int J Syst Evol Microbiol. 2019;69(11):3399–404. doi: 10.1099/ijsem.0.003630 31380735

[60] Wu X-T, He Y-Q, Li G-X, Xiao H, Dai X-R, Yang M-R, et al. Genome Sequence of Sulfide-Dependent Denitrification Bacterium Thermomonas sp. Strain XSG, Isolated from Marine Sediment. Microbiol Resour Announc. 2021;10(15):e00057-21. doi: 10.1128/MRA.00057-21 33858918 PMC8050960

[61] Khan ST, Hiraishi A. Diaphorobacter nitroreducens gen nov, sp nov, a poly(3-hydroxybutyrate)-degrading denitrifying bacterium isolated from activated sludge. J Gen Appl Microbiol. 2002;48(6):299–308. doi: 10.2323/jgam.48.299 12682868

[62] Kim S-J, Moon J-Y, Ahn J-H, Weon H-Y, Hong S-B, Seok S-J, et al. Diaphorobacter aerolatus sp. nov., isolated from air, and emended description of the genus Diaphorobacter. Int J Syst Evol Microbiol. 2014;64(Pt 2):513–7. doi: 10.1099/ijs.0.051060-0 24105945

[63] Kalyuhznaya MG, Martens-Habbena W, Wang T, Hackett M, Stolyar SM, Stahl DA, et al. Methylophilaceae link methanol oxidation to denitrification in freshwater lake sediment as suggested by stable isotope probing and pure culture analysis. Environ Microbiol Rep. 2009;1(5):385–92. doi: 10.1111/j.1758-2229.2009.00046.x 23765891

[64] Beck DAC, McTaggart TL, Setboonsarng U, Vorobev A, Kalyuzhnaya MG, Ivanova N, et al. The expanded diversity of methylophilaceae from Lake Washington through cultivation and genomic sequencing of novel ecotypes. PLoS One. 2014;9(7):e102458. doi: 10.1371/journal.pone.0102458 25058595 PMC4109929

[65] Bosch J, Lee K-Y, Jordan G, Kim K-W, Meckenstock RU. Anaerobic, nitrate-dependent oxidation of pyrite nanoparticles by Thiobacillus denitrificans. Environ Sci Technol. 2012;46(4):2095–101. doi: 10.1021/es2022329 22142180

[66] Haaijer SCM, Lamers LPM, Smolders AJP, Jetten MSM, Op den Camp HJM. Iron Sulfide and Pyrite as Potential Electron Donors for Microbial Nitrate Reduction in Freshwater Wetlands. Geomicrobiol J. 2007;24:391–401.

[67] Dimroth P, Schink B. Energy conservation in the decarboxylation of dicarboxylic acids by fermenting bacteria. Arch Microbiol. 1998;170(2):69–77. doi: 10.1007/s002030050616 9683642

[68] Pasalari H, Gholami M, Rezaee A, Esrafili A, Farzadkia M. Perspectives on microbial community in anaerobic digestion with emphasis on environmental parameters: A systematic review. Chemosphere. 2021;270:128618. doi: 10.1016/j.chemosphere.2020.128618 33121817

[69] Park CM, Novak JT. The effect of direct addition of iron(III) on anaerobic digestion efficiency and odor causing compounds. Water Sci Technol. 2013;68(11):2391–6. doi: 10.2166/wst.2013.507 24334887

[70] Zandvoort MH, van Hullebusch ED, Fermoso FG, Lens PNL. Trace Metals in Anaerobic Granular Sludge Reactors: Bioavailability and Dosing Strategies. Engineering in Life Sciences. 2006;6(3):293–301. doi: 10.1002/elsc.200620129

[71] Tilman D, Knops J, Wedin D, Reich P, Ritchie M, Siemann E. The influence of functional diversity and composition on ecosystem processes. Science. 1997;277:1300–2.

[72] Chao A, Chiu CH, Villéger S, Sun IF, Thorn S, Lin YC. An attribute‐diversity approach to functional diversity, functional beta diversity, and related (dis)similarity measures. Ecol Monogr. 2019;89:e01343.

[73] Findlay AJ. Microbial impact on polysulfide dynamics in the environment. FEMS Microbiol Lett. 2016;363(11):fnw103. doi: 10.1093/femsle/fnw103 27190288

[74] Schippers A, Jørgensen BB. Biogeochemistry of pyrite and iron sulfide oxidation in marine sediments. Geochimica et Cosmochimica Acta. 2002;66(1):85–92. doi: 10.1016/s0016-7037(01)00745-1

[75] Liu T, Hu Y, Chen N, He Q, Feng C. High redox potential promotes oxidation of pyrite under neutral conditions: Implications for optimizing pyrite autotrophic denitrification. J Hazard Mater. 2021;416:125844. doi: 10.1016/j.jhazmat.2021.125844 33878651

[76] Hu Y, Wu G, Li R, Xiao L, Zhan X. Iron sulphides mediated autotrophic denitrification: An emerging bioprocess for nitrate pollution mitigation and sustainable wastewater treatment. Water Res. 2020;179:115914. doi: 10.1016/j.watres.2020.115914 32413614

[77] Bosch J, Meckenstock RU. Rates and potential mechanism of anaerobic nitrate-dependent microbial pyrite oxidation. Biochem Soc Trans. 2012;40(6):1280–3. doi: 10.1042/BST20120102 23176468

[78] Vaclavkova S, Schultz-Jensen N, Jacobsen OS, Elberling B, Aamand J. Nitrate-controlled anaerobic oxidation of pyrite by thiobacillus cultures. Geomicrobiol J. 2015;32:412–9.

[79] Torrentó C, Cama J, Urmeneta J, Otero N, Soler A. Denitrification of groundwater with pyrite and Thiobacillus denitrificans. Chem Geol. 2010;278:80–91.

[80] Garber AI, Nealson KH, Okamoto A, McAllister SM, Chan CS, Barco RA, et al. FeGenie: A Comprehensive Tool for the Identification of Iron Genes and Iron Gene Neighborhoods in Genome and Metagenome Assemblies. Front Microbiol. 2020;11:37. doi: 10.3389/fmicb.2020.00037 32082281 PMC7005843

[81] Mouillot D, Graham NAJ, Villéger S, Mason NWH, Bellwood DR. A functional approach reveals community responses to disturbances. Trends Ecol Evol. 2013;28(3):167–77. doi: 10.1016/j.tree.2012.10.004 23141923

[82] Norberg J, Swaney DP, Dushoff J, Lin J, Casagrandi R, Levin SA. Phenotypic diversity and ecosystem functioning in changing environments: a theoretical framework. Proc Natl Acad Sci U S A. 2001;98(20):11376–81. doi: 10.1073/pnas.171315998 11535803 PMC58737

[83] Schoepfer VA, Bernhardt ES, Burgin AJ. Iron clad wetlands: Soil iron‐sulfur buffering determines coastal wetland response to salt water incursion. J Geophys Res Biogeosci. 2014;119:2209–19.

[84] Candry P, Abrahamson B, Stahl DA, Winkler M-KH. Microbially mediated climate feedbacks from wetland ecosystems. Glob Chang Biol. 2023;29(18):5169–83. doi: 10.1111/gcb.16850 37386740

[85] van den Berg EM, Boleij M, Kuenen JG, Kleerebezem R, van Loosdrecht MCM. DNRA and Denitrification Coexist over a Broad Range of Acetate/N-NO3- Ratios, in a Chemostat Enrichment Culture. Front Microbiol. 2016;7:1842. doi: 10.3389/fmicb.2016.01842 27933040 PMC5121219

[86] Arkin AP, Cottingham RW, Henry CS, Harris NL, Stevens RL, Maslov S. KBase: The United States Department of Energy Systems Biology Knowledgebase. Nat Biotechnol. 2018;36:566–9. 29979655 10.1038/nbt.4163 PMC6870991

[87] Bolger AM, Lohse M, Usadel B. Trimmomatic: a flexible trimmer for Illumina sequence data. Bioinformatics. 2014;30(15):2114–20. doi: 10.1093/bioinformatics/btu170 24695404 PMC4103590

[88] Jain M, Olsen HE, Paten B, Akeson M. The Oxford Nanopore MinION: delivery of nanopore sequencing to the genomics community. Genome Biol. 2016;17(1):239. doi: 10.1186/s13059-016-1103-0 27887629 PMC5124260

[89] Antipov D, Korobeynikov A, McLean JS, Pevzner PA. hybridSPAdes: an algorithm for hybrid assembly of short and long reads. Bioinformatics. 2016;32(7):1009–15. doi: 10.1093/bioinformatics/btv688 26589280 PMC4907386

[90] Nurk S, Meleshko D, Korobeynikov A, Pevzner PA. metaSPAdes: a new versatile metagenomic assembler. Genome Res. 2017;27(5):824–34. doi: 10.1101/gr.213959.116 28298430 PMC5411777

[91] Gurevich A, Saveliev V, Vyahhi N, Tesler G. QUAST: quality assessment tool for genome assemblies. Bioinformatics. 2013;29(8):1072–5. doi: 10.1093/bioinformatics/btt086 23422339 PMC3624806

[92] Wu Y-W, Simmons BA, Singer SW. MaxBin 2.0: an automated binning algorithm to recover genomes from multiple metagenomic datasets. Bioinformatics. 2016;32(4):605–7. doi: 10.1093/bioinformatics/btv638 26515820

[93] Parks DH, Imelfort M, Skennerton CT, Hugenholtz P, Tyson GW. CheckM: assessing the quality of microbial genomes recovered from isolates, single cells, and metagenomes. Genome Res. 2015;25(7):1043–55. doi: 10.1101/gr.186072.114 25977477 PMC4484387

[94] Langmead B, Salzberg SL. Fast gapped-read alignment with Bowtie 2. Nat Methods. 2012;9(4):357–9. doi: 10.1038/nmeth.1923 22388286 PMC3322381

[95] Chaumeil P-A, Mussig AJ, Hugenholtz P, Parks DH. GTDB-Tk: a toolkit to classify genomes with the Genome Taxonomy Database. Bioinformatics. 2019. doi: 10.1093/bioinformatics/btz848 PMC7703759 31730192

[96] Price MN, Dehal PS, Arkin AP. FastTree 2--approximately maximum-likelihood trees for large alignments. PLoS One. 2010;5:e9490. 10.1371/journal.pone.0009490 PMC2835736 20224823

[97] Hyatt D, Chen G-L, Locascio PF, Land ML, Larimer FW, Hauser LJ. Prodigal: prokaryotic gene recognition and translation initiation site identification. BMC Bioinformatics. 2010;11:119. 20211023 10.1186/1471-2105-11-119 PMC2848648

[98] Huerta-Cepas J, Forslund K, Coelho LP, Szklarczyk D, Jensen LJ, von Mering C, et al. Fast Genome-Wide Functional Annotation through Orthology Assignment by eggNOG-Mapper. Mol Biol Evol. 2017;34(8):2115–22. doi: 10.1093/molbev/msx148 28460117 PMC5850834

[99] Huerta-Cepas J, Szklarczyk D, Heller D, Hernández-Plaza A, Forslund SK, Cook H, et al. eggNOG 5.0: a hierarchical, functionally and phylogenetically annotated orthology resource based on 5090 organisms and 2502 viruses. Nucleic Acids Res. 2019;47(D1):D309–14. doi: 10.1093/nar/gky1085 30418610 PMC6324079

[100] Buchfink B, Xie C, Huson DH. Fast and sensitive protein alignment using DIAMOND. Nat Methods. 2015;12(1):59–60. doi: 10.1038/nmeth.3176 25402007

[101] Huang L, Zhang H, Wu P, Entwistle S, Li X, Yohe T, et al. dbCAN-seq: a database of carbohydrate-active enzyme (CAZyme) sequence and annotation. Nucleic Acids Res. 2018;46(D1):D516–21. doi: 10.1093/nar/gkx894 30053267 PMC5753378

[102] Kanehisa M, Sato Y, Morishima K. BlastKOALA and GhostKOALA: KEGG tools for functional characterization of genome and metagenome sequences. J Mol Biol. 2016;428:726–31. 26585406 10.1016/j.jmb.2015.11.006

[103] Shaffer M, Borton MA, McGivern BB, Zayed AA, La Rosa SL, Solden LM, et al. DRAM for distilling microbial metabolism to automate the curation of microbiome function. Nucleic Acids Res. 2020;48(16):8883–900. doi: 10.1093/nar/gkaa621 32766782 PMC7498326

[104] Danecek P, Bonfield JK, Liddle J, Marshall J, Ohan V, Pollard MO, et al. Twelve years of SAMtools and BCFtools. Gigascience. 2021;10(2):giab008. doi: 10.1093/gigascience/giab008 33590861 PMC7931819

